# Deciphering Possible Association of Risk Factors for Dental Caries in Pakistani Population

**DOI:** 10.1155/2018/2842350

**Published:** 2018-02-28

**Authors:** Shahida Maqsood, Hasan Baber, Zia Abbas, Javeria Ali Khan, Muznah Khalid

**Affiliations:** Dow University of Health Sciences, Karachi, Pakistan

## Abstract

Obesity is a state of abnormal accumulation of fat in adipose tissues of the body to the level that one's health is adversely compromised. Tripathi et al. state (according to WHO) that obesity is now considered the fifth leading cause of mortality in the world. Caries is a multifactorial disease and one of the major oral health issues of the modern era affecting people around the globe. Rise in dental caries has been observed in developing countries as a result of factors including increased intake of carbohydrates. The present study aims for assessing the association of DMFT with BMI, age, and gender. This study was conducted in the dental OPD of the Dow University of Health Sciences, Karachi, from February 2016 till January 2017. A custom-made interview-based questionnaire was used to assess BMI, DMFT, and sociodemographics. The sample size was kept at 385. Age was reported as a strong predictor (*R* ^2^ 0.641) of DMFT followed by BMI and gender as the weakest predictors. Age and BMI had statistically significant association with DMFT scores, which shows that diet patterns may affect general health. High caloric intake over long periods affects BMI and also oral health.

## 1. Introduction

According to WHO, obesity is a state of abnormal accumulation of fat in adipose tissues of the body to the level that one's health is adversely compromised [1]. Alm et al. and Tripathi et al. state (*according to WHO*) that obesity is now considered the fifth leading cause of mortality in the world [2]. Reports suggest that the prevalence of obesity in the last two decades has doubled in developed and developing countries [3]. Association of obesity with certain systemic diseases such as type 2 diabetes, hypertension, and cerebrovascular diseases have been strongly reported in literature [3–7].

A comprehensive study of the National Health and Nutrition Examination Survey (NHANES) between 1999 and 2004 reported among individuals of 20–34 years had 85.58% DMFT, 35–50 years had 94.30% DMFT, and 50–64 years had 95.62% DMFT [8]. This suggests that DMFT score increases with age. In recent years, due to increase in refined, fat diet, and sedentary lifestyles, obesity has increased in South Asia. As highlighted by Amin et al., one major factor of obesity is change in regional diet and its quantity and quality [9]. Recent studies have demonstrated an increase in the consumption of animal and refined foods with reduction in the intake of vegetables [10].

Caries is the main cause of dental pain and loss of tooth [11]. It accounts for one of the major oral health-related diseases especially among younger individuals. Due to caries being so widespread globally, it is present among both genders, all age groups, and among all cultures and socioeconomic classes [12]. Dental caries not only results in financial burden but also has a major impact on the quality of life of an affected individual. Treatment of dental caries is also expensive. According to a WHO report, caries is the 2nd most costly disease in Australia followed by cardiovascular diseases [13]. Progression in caries,prevention, and treatment regimens are achieved via scientific research. In the last ten years, a reduction in dental caries has been observed in developed nations [14, 15]. Contrary to this, a rise in dental caries has been observed in developing countries as a result of factors including an increase in the intake of carbohydrates and high-sugar diet in the form of desserts, increase in people with low-socioeconomic status, lack of education, and basic health-care services [16]. A number of studies have been documented assessing the association of BMI and dental caries with conflicting results. The present study aims for assessing association of DMFT with BMI, age, and gender.

Our null hypothesis is that BMI, age, and gender are not associated with DMFT (caries), and BMI and age are weak predictors of DMFT.

Our alternate hypothesis is that BMI, age, and gender are associated with DMFT (caries), and BMI and age are strong predictors of DMFT.

## 2. Methodology

The study was conducted in the dental OPD of the Dow University of Health Sciences, Karachi, between February 2016 and January 2017. No issues were observed during routine patient care, as this study had no direct involvement regarding patient treatment or intervention. A custom-made questionnaire was used to assess BMI, DMFT, and sociodemographics of participants via an interview available here). Consent was obtained from all participants before the study. Keeping the confidence interval at 95% with standard error of mean at 0.5%, the calculated sample size (considering annual patient flow in OPD) was 385. Participants within the age group of 14 to 65 years old were included. Edentulous patients were excluded. Patients having any communicable disease or any psychotic disorder or patients who have conditions restricting them to a very strict diet regimen, patients with radiation therapy, and pregnant women were also excluded.

BMI was categorized as underweight (BMI < 18.5), normal (BMI 18.5–24.9), overweight (BMI 25–30), and obese (BMI > 30). Participants were inquired regarding their eating habits, frequency of eating, consumption of refined sugars, snacks and fast foods, physical activity, and tooth-brushing habits.

Caries prevalence was assessed via standard criteria set up by the WHO, the decayed, missing, and filled teeth (DMFT) index. The index determines the total number of dentition, surfaces of teeth having caries, and missing or had restorative procedures. Clinical dental examination was undertaken by house officers specially trained in dental OPD, with the use of natural sunlight or a source of illumination wherever needed. In accordance with infection control guidelines, new cap, mask, and gloves were used for each patient. Questions regarding lifestyle were assessed by the time spent on watching TV, use of computers, playing games on consoles or computers, and use of smart phones for the purpose of entertainment.

## 3. Statistical Analysis

Data were analyzed using the Statistical Package for Social Science software, version 20.0 (SPSS, Chicago, IL, USA). The chi-square test was used to study the relationship between variables and to compare means. *p* value < 0.05 was considered statistically significant. Linear logistic regression analysis was used to determine the degree of association between obesity and dental caries and other variables.

## 4. Results

**Figure 1:**
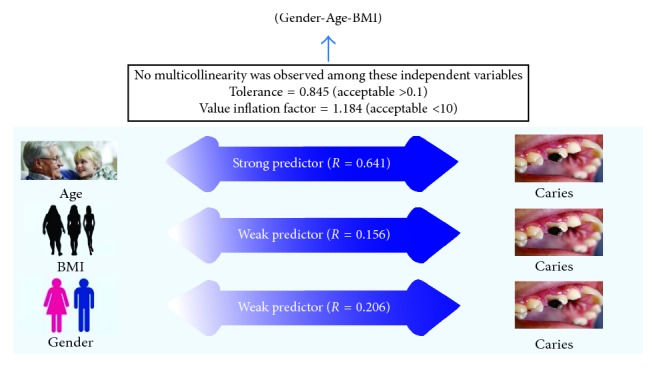


A total of 385 patients were enrolled in the study. The mean age was 24.60 years, and the mean DMFT was 3.01. Young females constitute the highest number of participants. Results are tabulated in Figure 1 and Tables 1 [Table tab2]–3.

With regard to possible associations between variables, age and BMI had demonstrated statistically significant association with DMFT scores. According to our results, *R* ^2^ of 64.1% variance in age can be a strong predictor of DMFT score, while BMI (15.6%) and gender (20.6%) variance is not good enough in predicting DMFT scores of an individual.

## 5. Discussion

Both dental caries and BMI are related to diet-related health outcomes, and the association between the two is not surprising. Previously, past studies have also reported the association between these two variables [17]. Since the mid-1990s, there have been drastic changes in lifestyle and diet, probably due to the increased usage of food having rich calories and intake of carbohydrate drinks and foods. This can be one of the etiological factors of rise in obesity and dental caries [17–19]. Obesity has seen a rise in the US and Europe [20]. According to Eurostat's statistics, a report published in 2014 states that European Union states as of 2014 had staggering 51.4% of population above 18 years. A similar report of NHANES (National Health and Nutrition Survey) in a 2012 survey states that US individuals above 20 years having BMI of 30 plus [21]. The present study had less overweight individuals than those with normal BMI; thus, the majority of high DMFT score was among normal weight individuals; probably here factors determining caries are others such as poor oral hygiene, lack of education, and so on. The result was as expected as according to Wan Siang et al. Asian countries have some of the lowest prevalences of overweight and obesity worldwide, with Vietnam and India having the lowest with 1.7% and 1.9%, respectively [22].

Older adults demonstrated highest number of DMFT scores. A survey by the National Institute of Dental and Craniofacial Research between 1999 and 2004 reported the age group of 20–34 years constituting the highest number of decayed untreated teeth, but with lowest DMFT score. The current study also demonstrated a similar trend.

Sheiham and Sabbah [21] stated that levels of caries follow a predictable trend, provided stable environmental conditions and absence of any effective interventions [23]. In the present study, a large difference is observed in DMFT scores between adults and youngsters. This is in-line with similar findings in an Iranian study where higher age reported increase in caries experience [24]. Similar results were also reported in the northern and southern Indian study [25, 26]. High DMFT scores among adults can also be accounted due to neglect from young individuals regarding their oral care, and it is only in later stages of life that carious lesion progresses to a significant level that an individual seeks dental assistance.

Caries is a multifactorial disease. Numbers of factors are responsible for initiation of dental caries such as composition and frequency of diet, socioeconomic status, salivary immunoglobulins, and bacterial and fluoride intake. Due to multiple etiologies, the study of dental caries has become complex. Apart from this, obesity and dental caries are complex conditions with etiological factors such as genetics, biological, behavioral, and environmental. BMI is used widely as a measurement tool for obesity due to the fact that it relates the height of a person with respect to his weight. It is also a tool for nutritional status indicator. Thus, it proves the fact that both dental caries and BMI are used as measurement tools for diet-related health outcomes. An association amongst them is not surprising.

Apart from this, malnourishment is also one of the etiologies for dental caries. Protein deficiency/energy loss leads to energy-protein malnourishment, reduction in the flow of saliva, formation of calculus, increase in carious lesions, and growth reduction. Studies have reported malnourishment in young adults predisposing to increase propensity to dental caries and salivary hypofunction [27].

The present study demonstrated statistically significant association between BMI and DMFT. A study by Elkhodary et al. reported significant association between more weight gain and dental caries [26]. Possible explanation can be the fact that obese individuals tend to consume high levels of soda and other sugary drinks and foods which by nature are obesogenic and cariogenic. Modéer et al. gave an opinion that overweight individuals have high caries risk as a result of reduction in the salivary flow rate, which itself is associated with protein-deficient malnourishment [27]. Contrary to these reported studies, Macek and Mitola reported the absence of any statistically significant association between age, BMI, and dental caries [28]. This clearly suggests that dental caries having multifactorial causes and associations can be as a result of similar trends observed in a specific population studied.

## 6. Conclusion

Although the exact nature of these associations is complex and can involve many other confounding variables that otherwise could affect the final outcome, the study has demonstrated that dental caries is influenced by factors such as age and BMI. Further studies on a much larger and diverse scale with factors such as socioeconomic status and dietary patterns will aid in further clarifying the risk factors of dental caries.

## Figures and Tables

**Table 1:** 

Age groups	*p*=0.001
	DMFT
Teenagers (14–19)	28
Young adults (20–40)	19
Adults (41–65)	338

**Table 2 tab2:** 

Gender	*p*=0.301
	DMFT
Male	92
Female	293

**Table 3:** 

BMI	*p*=0.003
	DMFT
Underweight	42
Normal	298
Overweight	45
Obese	27

## Abbreviations

DMFT: Decayed missing filled tooth
BMI: Body mass index
WHO: World Health Organization
OPD: Outpatient department
NHANES: National Health and Nutrition Examination Survey.

## Supplementary Materials

Supplementary Materials “BMI DMFT Questionnaire” used in this study.
