# Supplementary material for: Deciphering Possible Association of Risk Factors for Dental Caries in Pakistani Population
Source: Int J Dent. 2018 Feb 28;2018:2842350. doi: 10.1155/2018/2842350 (PMC5851031; doi:10.1155/2018/2842350)
Supplement: Supplementary Materials — “BMI DMFT Questionnaire” used in this study. [file 2842350.f1.pdf]

Do you give consent to take part in this study Yes No

Age: Gender: Male Female

Sugers/Beverage intake Questions

Sweetened suger drinks	# Tsp of Suger	1 time /week	2-3 times/ week	1 time/ day	2time /day	3 times /day	<6 fl oz (3/4 cup)	8 fl oz (1 cup)	12 fl oz (1 ½ cup)	16 fl oz (2 cups)	More than 20 Fl Oz (2 ½ cups)
Whole Milk											
Soft drinks regular											
Sweetened Tea/Coffee											
Energy/Sports Drinks											
Fruit Juices											

Fat/Sweets Intake Questions

Please Tick your Response	Times Per Week				
How Many Times a week would you	Never	Less than once	1 - 2	3 - 5	6 or more
Eat Fried Food					
French fries					
Eat pasteries,cakes,seet biscuits or Bun					
Eat chocolate, chocolate biscuits or sweet snack bars					
Eat potato chips,corn chips or something similar					
Have ice cream or dessert					
How is your meal cooked	I eat meat occasionally/Never	Grilled/Roasted (No Oil)	Grilled roasted (With Oil)	Stewed (Kari)	Fried
Total (add ticks)					

**Multiply total by:**	**X 0**	**X 1**	**X 2**	**X 3**	**X 4**
**Preliminary Score (Add preliminary scores together)**					

Scoring:

1: 0-10 (Low fat) **2**:11-17 (Low to moderate fat) **3**:18-25 (Moderate Fat) **4**:26-39 (Moderate to High fat) **5**: 40+ (High Fat)

How often do you do physical exercise?

1:Daily **2**: Once a week **3**:Twice a week **4**:Thrice a week **5**: More than thrice a week 6:Never

BMI related questions

What is your height?: What is your weight?:

BMI =

DMFT Assessment:

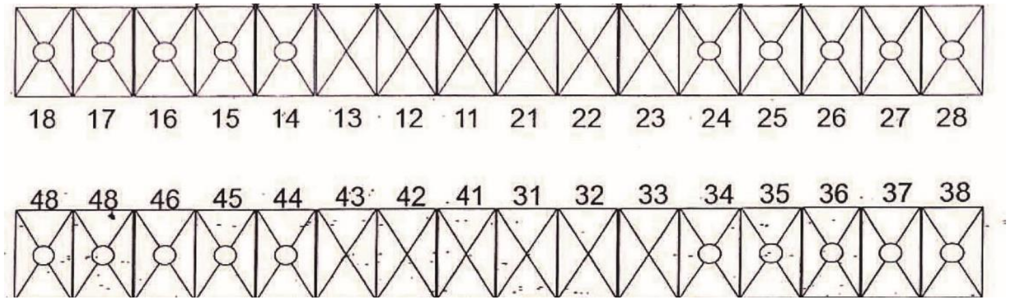

D: Decayed F: Filled

Decayed (Total)	Filled (Total)
